# Acute effects of different Tai Chi practice protocols on cardiac autonomic modulation

**DOI:** 10.1038/s41598-024-56330-9

**Published:** 2024-03-06

**Authors:** Dejian Duan, Dong Wang, Haojie Li, Wenbo Li, Dong Wu

**Affiliations:** 1 China Wushu School, Beijing Sport University, Beijing, 100084 China; 2 Wushu and Dance School, Shenyang Sports University, Shenyang, 110102 China; 3 School of Physical Education and Exercise, Beijing Normal University, Beijing, 100875 China; 4 China University of Geosciences (Beijing), Beijing, 100083 China

**Keywords:** Tai Chi, Heart rate variability, Intensity, University student, Human behaviour, Psychology, Cardiology, Health care, Health occupations, Medical research

## Abstract

Tai Chi serves as an effective exercise modality for enhancing autonomic regulation. However, a majority of existing studies have employed the single routine (SR) protocol as the basis for health interventions. The extent to which the gong routine application (GRA) protocol achieves similar levels of exercise load stimulation as traditional single practice routines remains uncertain. Therefore, this study the distinct characteristics of autonomic load stimulation in these different protocols, thus providing a biological foundation to support the development of Tai Chi health promotion intervention programs. we recruited a cohort of forty-five university students to participate in the 15 min GRA protocol and SR protocol. We collected heart rate and heart rate variability indicators during periods of rest, GRA protocol, and SR protocol utilizing the Polar Scale. Additionally, we assessed the mental state of the participants using the BFS State of Mind Scale. In summary, the autonomic load is lower in the GRA protocol compared to the SR protocol, with lower sympathetic activity but higher parasympathetic activity in the former. Results are specific to college students, additional research is necessary to extend support for frail older adults. It is advised to incorporate GRA protocol alongside SR protocol in Tai Chi instruction. This approach is likely to enhance Tai Chi skills and yield greater health benefits.

## Introduction

Tai Chi, a form of martial art originating in China over four centuries ago, has gained worldwide popularity for its numerous health benefits^1,2^. This moderate-intensity aerobic exercise combines deliberate deep breathing with slow, meditative movements, promoting meditation and concentration^3,4^. Tai Chi emphasizes upper-body movements while maintaining a squatting posture in the lower body^5^. These movements, referred to as "form" are performed sequentially to create a "routine"^6^. A complete Tai Chi set comprises 24 distinct postures, allowing practitioners to adapt their practice to their individual fitness needs and abilities^7^. This versatility makes Tai Chi suitable for individuals of all ages and health conditions^8^. Notably, Tai Chi has proven to be safe not only for those in good health but also for individuals with chronic illnesses^9^.

The global appeal of Tai Chi can be attributed to its demonstrated health advantages. Studies have shown that Tai Chi effectively alleviates symptoms associated with conditions like Parkinson's disease^10^ and cognitive impairment^11^. It also reduces anxiety and stress levels while enhancing overall strength, balance, and flexibility^12,13^. Tai Chi contributes to improved aerobic capacity, positively impacts cardiovascular risk factors, and lowers blood pressure^14^. Moreover, its versatility allows it to be practiced individually or in group settings, making it accessible to individuals with busy lifestyles and fostering social engagement^15^. Furthermore, Tai Chi requires no special equipment and can be enjoyed by virtually anyone, anywhere^16^.

The cardiac autonomic nervous system comprises sympathetic and parasympathetic nerves, which innervate the atria and ventricles through post-ganglionic fibers, often passing through the stellate ganglion^17^. Parasympathetic nerves primarily originate from the nucleus of the vagus nerve, mainly innervating the atria, and are commonly referred to as the vagus nerve due to their presence within it^18^. The rhythmic heartbeat results from the balanced regulation of both sympathetic and parasympathetic nerves^19^. This coordination and antagonism between parasympathetic and sympathetic nerves are central to cardiac autonomic regulation^20^. Research has highlighted the close association between dysregulated autonomic function and various diseases, particularly cardiovascular ailments and the risk of sudden death^21^. Heart Rate Variability (HRV) analysis, a non-invasive method, serves as a robust means of assessing cardiac autonomic regulation and its dynamic activity changes^22^. HRV parameters offer insights into the sympathovagal balance in the body and the extent of parasympathetic influence on sympathetic nerves, making it valuable for prognosis in various clinical scenarios, including acute myocardial infarction, coronary heart disease (CHD), and heart failure^23^.

Deep breathing and aerobic exercise are effective strategies for enhancing parasympathetic nerve activity, consequently improving Heart Rate Variability (HRV)^24,25^. Tai Chi uniquely combines these characteristics, positioning it as an effective exercise modality for HRV enhancement^26^. Existing studies have predominantly focused on 24-form Tai Chi single-practice protocol for health interventions. The SR protocol allows practitioners to roughly understand the fundamentals of Tai Chi skills and master its basic forms. Nonetheless, the SR protocol poses challenges for practitioners in comprehending the functions of individual Tai Chi movements and the overall movement pattern, potentially hindering a deep mastery of Tai Chi skills. Traditional Tai Chi entails systematic training, encompassing standing poles (gong fa), routines, and self-defense applications. Emphasis in Traditional Tai Chi training lies in introducing standing poles, exploring methods through routines, and uncovering truths through self-defense applications. The Standing Pole component primarily focuses on cultivating various static and dynamic fundamental Tai Chi movements, establishing a foundation for subsequent routine practice. The routine part comprises individual Tai Chi movement exercises and multiple movement combinations, constituting the essence of Tai Chi practice. The self-defense application part primarily emphasizes the practical implementation of both individual movements and combinations within routines, serving as a test of practitioners' mastery of routine skills. The GRA protocol assists practitioners in mastering the distinctive movement pattern characterized by synchronous movement of the upper and lower limbs, propelled by the core area. It enhances appreciation for the application of each Tai Chi movement and stimulates practitioners' interest in Tai Chi. The GRA protocol effectively addresses certain limitations inherent in the single routine by seamlessly integrating standing poles, routines, and self-defense applications. This approach serves to enhance the practitioner's accuracy in routine skills and fosters a deeper mastery of Tai Chi skills. However, the evolving GRA protocol, designed to aid practitioners in mastering Tai Chi skills, raises questions regarding its ability to induce exercise load stimulation similar to that of traditional SR protocol. In this study, we aimed to compare the 24-form Tai Chi GRA protocol with the SR protocol, particularly with regard to heart rate variability. Our purpose was to elucidate the autonomic load stimulation characteristics of these different protocols, offering biological evidence to support the development of Tai Chi health-promotion intervention programs.

## Methods

### Calculation of sample size

The required sample size for the study was pre-estimated using G-power 3.1.9.7 software.The study set Partial ŋ2 to 0.06, resulting in an effect size of 0.2526456. ɑ err pob, Power (1-βerr prob),Corr among rep measures, and Nonsphericity correction were set to 0.05, 0.96,0.5, and 1, respectively. The results showed that 45 subjects were needed for the trial.

### Participants

All participants provided written informed consent and understood the experimental process and purpose. All methods in this study were carried out following the Declaration of Helsinki's relevant guidelines and regulations, and this study was reviewed and approved by the ethical review of the Beijing Sport University (NO.2023161H), and informed consent was obtained from all subjects.

For the purpose of this experiment, a total of 45 college students specializing in martial arts set direction were recruited. Inclusion criteria consisted of the following: (1) Negative responses to all seven questions on the American College of Sports Medicine Physical Activity Readiness Questionnaire (PAR-Q). (2) No abnormalities detected on the electrocardiogram (ECG).

Exclusion criteria were defined as follows: (1) Chronic illnesses or a history of major illnesses. (2) Experiences of sports-related injuries or fractures within the past three years. (3) Engagement in high-intensity physical activities (e.g., basketball, running, skiing, etc.) within 72 h preceding the test.

All recruited students held a minimum status of Martial Art National Level 2 Athlete or higher. They demonstrated proficiency in executing the 24-form Tai Chi SR protocol and had completed a minimum of one semester, equivalent to 32 h, of classroom training in the GRA protocol. Furthermore, they exhibited the capability to execute all elements of the 24-form Tai Chi GRA protocol to a satisfactory standard. The participant group comprised 26 males and 19 females, as outlined in Table 1.

**Table 1:** List of basic conditions of Tai Chi practitioners.

Gender	Age (years)	Height (cm)	Weight (kg)	BMI (kg/m^2^)
Male (n = 26)	20.52 ± 2.24	176.68 ± 5.53	68.98 ± 6.22	22.08 ± 1.59
Female (n = 19)	21.74 ± 2.16	162.09 ± 4.97	56.09 ± 5.80	21.34 ± 1.76

### Experimental procedure

The experiments were conducted daily between 9 a.m. and 12 p.m. The experiment took place in a serene and uninterrupted martial arts gym. Participants entered the martial arts gym, where their height and weight were measured before donning a Polar belt. All participants consistently wore a Polar during the entire data collection process. Heart rate variability data were concurrently gathered for 15 min during a quiet state, 15 min during the GRA protocol, and 15 min during the SR protocol. Subsequent to the GRA and SR protocols, participants promptly completed the BFS Mood Scale. To mitigate any potential sequential effects resulting from the different protocols, the 45 Tai Chi subjects were randomly assigned to two groups for data collection. The ABBA design employed in this study aims to minimize autonomic differences between the two protocols arising from sequential issues.

Group A (n = 23): This group underwent testing first in the GRA protocol and then in the SR protocol.

**Figure 1:**
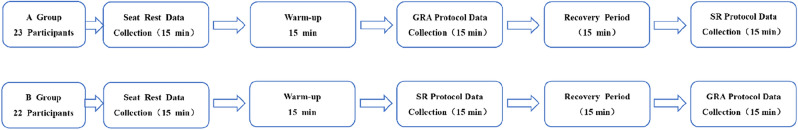
Schematic diagram of the experimental data collection process.

Group B (n = 22): Conversely, Group B participants were tested initially in the SR protocol and subsequently in the GRA protocol. For a visual representation of the experimental design, please refer to Fig. 1.

### Tai Chi exercise protocol

**Figure 2:**
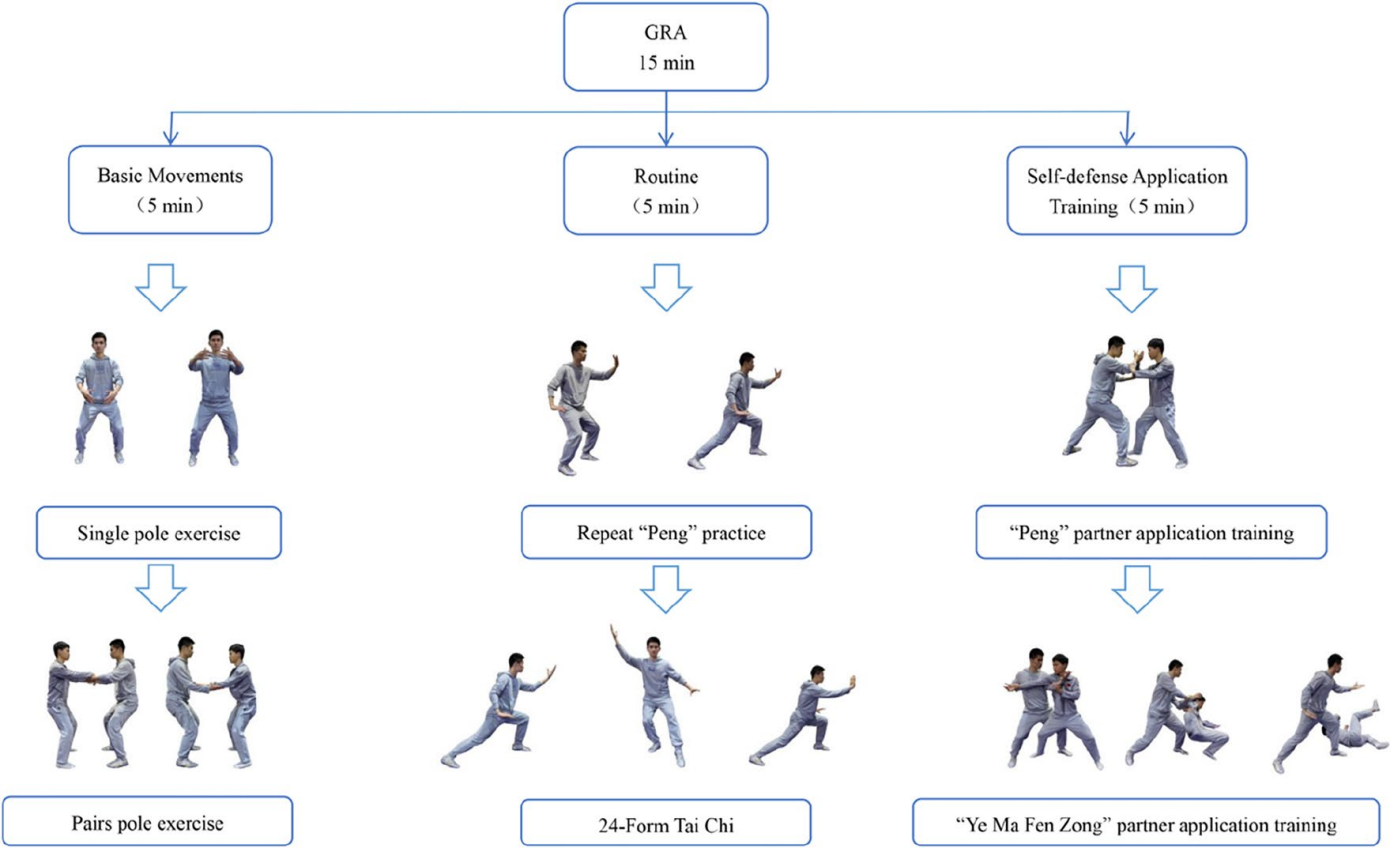
Schematic diagram of the 24-form Tai Chi's gong routine application protocol.

The two distinct Tai Chi protocols employed in this study are as follows: SR protocol: In this protocol, participants executed a sequence consisting of three repetitions of the 24-form Tai Chi routine. GRA protocol: This protocol was further divided into three practice sections, each lasting for 5 min. Basic Movements Section (Gong Fa/Zhan Zhuang): Focused on the development of foundational skills, particularly emphasizing standing pole exercise training. Routines Section: Comprised movements like the "Peng" movement and the 24-form Tai Chi routine. Self-defense Application Training Section: Participants engaged in paired practice, applying Tai Chi self-defense techniques in a cooperative manner. For a visual representation, please refer to Fig. 2.

### Evaluation of autonomic activity

Participants ensured optimal conditions by adhering to the following criteria the day before the test:

A minimum of 6 h of uninterrupted sleep. Abstention from smoking, alcohol consumption, staying up late, as well as the use of coffee or stimulant drugs. Participants refrained from eating and maintained emotional stability for at least one hour prior to the test. Autonomic activity was assessed using the HRV method. RR intervals (iRR) were obtained using the Polar Team 2 Heart Rate Monitor. Kubios software was employed to calculate R-R intervals and associated variability from the recorded data. The following time-domain indicators were selected for analysis: The Square Root of The Mean Squared Differences of Successive NN Intervals (RMSSD): Low Frequency Power (LF), High Frequency Power (HF), Low Frequency Power to High Frequency Power ratio (LF/HF). The study standardized LF and HF values as LF (nu) and HF (nu) to facilitate between-group comparisons.

Mindfulness states of Tai Chi practitioners were evaluated using the BFS Mood Scale after GRA and SR protocol. The BFS Mood Scale was developed by Zerssen in 1970^27^. The BFS Mood Scale is a mindfulness measurement tool based on the theory of the two-dimensional components of mindfulness (positivity and negativity)^28^. The scale assesses changes in the subject's state of mind, prompting them to report their true feelings at the time. The scale comprises eight subscales, including Vitality, Pleasure, Thoughtfulness, Calmness, Anger, Excitability, Depression, and Inactivity. Each subscale comprises five questions, resulting in a total of 40 questions. All questions were randomly mixed and presented, and respondents provided ratings on a 5-point Likert scale, ranging from "not at all" to "completely". The BFS Mood Scale is user-friendly, requiring subjects approximately 5 min to complete. The Chinese version of the BFS Mood Scale was utilized for language consistency, aiming to facilitate subjects' completion. In 1997, the BFS Mood Scale was translated into Chinese, and validity tests were conducted^29^. The internal consistency reliability was computed to be 0.721 for the GRA positive state of mind and 0.705 for the GRA negative state of mind. For the SR positive state of mind, the internal consistency reliability was calculated to be 0.764, and for the SR negative state of mind, it was 0.864.

### Statistical analysis

Statistical analysis of the data was conducted using SPSS 23. Values conforming to a normal distribution were expressed as mean ± standard deviation (${\bar{\text{x}}}$ ± SD), while those not conforming were represented as median and interquartile range. Differences in heart rate and heart rate variability indicators among the quiet state, GRA protocol state, and SR protocol state of the Tai Chi subjects were assessed using repeated measures ANOVA. Variability tests among gong fa, routine, and self-defense application were examined using repeated measures ANOVA. To examine the variability of mindfulness indicators in the GRA protocol state and SR protocol state of the Tai Chi subjects, paired-sample *t* tests were applied. A significance level of *P* < 0.05 was set for all statistical analyses.

### Ethical review approval

This experiment was approved by the ethical review of the Beijing Sport University (NO.2023161H).

## Results

### Heart rate

**Figure 3:**
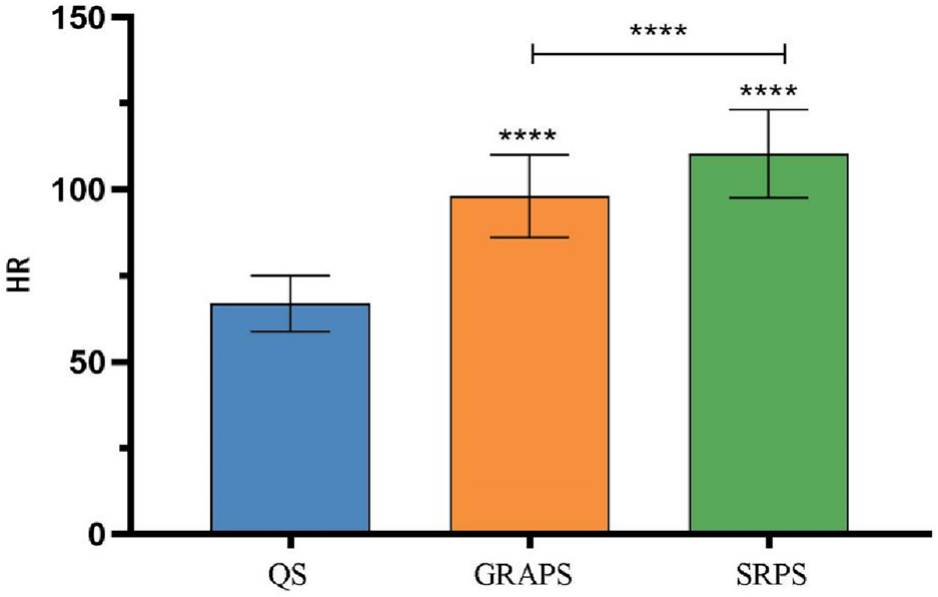
Schematic diagram of heart rate comparison between the state of 24-form Tai Chi’s GRA protocol and SR protocol. (Note: ****P < 0.05;the symbol "****" above each bar indicates a significant difference compared to the quiet state, *P* < 0.05;  the HR between the two groups is significantly different, *P* < 0.05.

As shown in Fig. 3**,** repeated-measures ANOVA was conducted to examine the heart rate indices of Tai Chi practitioners in various states. Mauchly's test of sphericity indicated a violation of the Huynh–Feldt condition (*P* < 0.05). Consequently, the Greenhouse–Geisser estimate was employed to correct for degrees of freedom. The correction revealed a significant main effect over time (*P* < 0.05).

Subsequent post hoc pairwise comparisons were conducted to further elucidate the distinctions between the GRA protocol and the SR protocol. A significant difference (*P* < 0.05) in heart rate was observed among the following states: Quiet State: 66.93 ± 8.05 beats/min, GRA protocol State: 98.09 ± 11.98 beats/min, SR protocol State: 110.42 ± 12.87 beats/min. Further analysis through post hoc pairwise comparisons revealed that the mean heart rate was significantly lower in the GRA protocol than in the SR protocol (*P* < 0.05).

### State of mind

As shown in Table 2, a paired-sample *t* test was conducted to compare the state of mind indicators between Tai Chi practitioners in the GRA protocol state and those in the SR protocol state. The results indicate significant differences in the following scores: Agonistic Score: GRA protocol (6.98 ± 2.03) versus SR protocol (6.30 ± 1.96), t(DF) = 2.345, *P* < 0.05. Active Score: GRA protocol (17.93 ± 4.55) versus SR protocol (16.60 ± 4.93), t(DF) = 2.657, *P* < 0.05. Calmness Score: GRA protocol (15.19 ± 3.23) versus SR protocol (16.53 ± 3.76), t(DF) = -2.519, *P* < 0.05.

**Table 2:** The comparison of the state of mind between 24-form Tai Chi’s GRA protocol and SR protocol.

	GRA	SR	T value	*P* value
Vitality	17.93 ± 0.69	16.60 ± 0.75	2.657	0.01*
Pleasure	16.58 ± 0.57	16.63 ± 0.56	− 0.093	0.926
Thoughtfulness	11.26 ± 0.55	11.95 ± 0.63	− 1.634	0.110
Calmness	15.19 ± 0.49	16.53 ± 0.57	− 2.519	0.016*
Anger	5.65 ± 0.22	5.53 ± 0.28	0.532	0.598
Excitability	6.98 ± 0.30	6.30 ± 0.30	2.345	0.024*
Depressive	5.50 ± 0.18	5.83 ± 0.26	− 1.573	0.123
Inactivity	6.28 ± 0.36	6.19 ± 0.29	0.213	0.832

*Means *P* < 0.05, there is a significant difference between the GAR protocol state and the SR protocol state.

### Time domain indicators of heart rate variability

**Figure 4:**
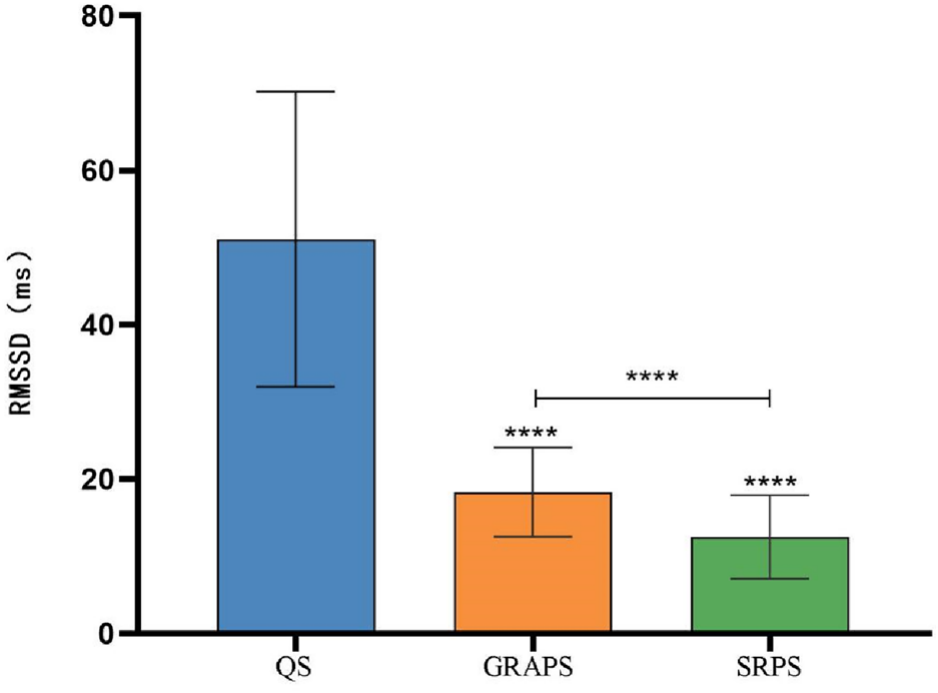
Schematic diagram of the RMSSD comparison between the state of 24-form Tai Chi’s GRA protocol and SR protocol. Note: ****P < 0.05;the symbol “****” above each bar indicates a significant difference compared to the quiet state, *P* < 0.05;  the RMSSD between the two groups is significantly different, *P* < 0.05.

As shown in Fig. 4, we conducted a repeated-measures ANOVA on the RMSSD of various states among Tai Chi practitioners. Mauchly's test of sphericity indicated a violation of the Huynh–Feldt condition (*P* < 0.05), necessitating the use of the Greenhouse-Gsiser estimate to correct for degrees of freedom. This correction revealed a significant main effect over time (*P* < 0.05).

Significant differences (*P* < 0.05) in RMSSD were observed among the following states: the quiet state (51.03 ± 19.12 ms), the GRA protocol state (18.31 ± 5.80 ms), and the SR protocol (12.50 ± 5.42 ms). Subsequently, a post hoc pairwise comparison was performed to delve deeper into the distinguishing characteristics between the GRA protocol state and the SR protocol state. The results underscored that the RMSSD in the GRA protocol state was significantly higher than that in the SR protocol state (*P* < 0.05).

### Frequency domain indicators of heart rate variability

**Figure 5:**
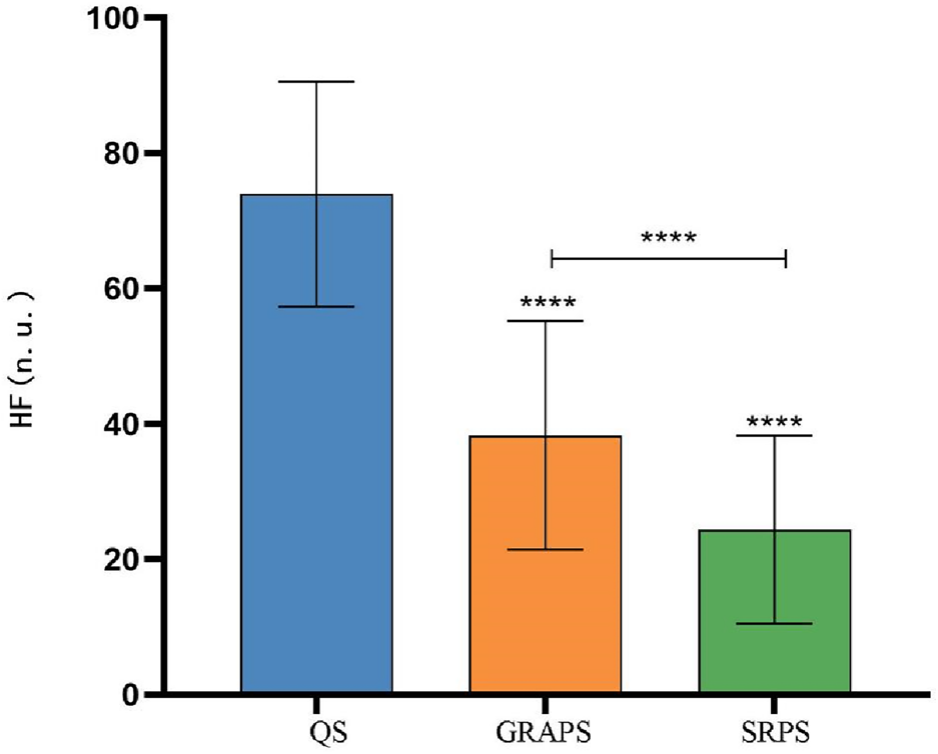
Schematic diagram of the HF (nu) comparison between the state of 24-form Tai Chi’s GRA protocol and SR protocol. Note: ****: *P* < 0.05;the symbol "****" above each bar indicates a significant difference compared to the quiet state, *P* < 0.05;  the HF(nu) between the two groups is significantly different, *P* < 0.05.

As shown in Fig. 5, the HF (nu) data collected from various states of Tai Chi practitioners underwent repeated measures ANOVA, and Mauchly's test of sphericity confirmed adherence to the Huynh–Feldt condition (*p* > 0.05), thus validating the results of the univariate ANOVA. These results revealed a significant main effect over time (*P* < 0.01).

Significant disparities (*P* < 0.05) in HF (nu) were observed among different states, namely, the quiet state (73.96 ± 16.63 ms), the GRA protocol state (38.32 ± 16.89 ms), and the SR protocol state (24.39 ± 13.94 ms). To further elucidate the distinctions between the GRA protocol state and the SR protocol state, a post hoc pairwise comparison was conducted. The outcomes demonstrated that HF (nu) was significantly higher in the GRA protocol state compared to the SR protocol state (*P* < 0.05).

**Figure 6:**
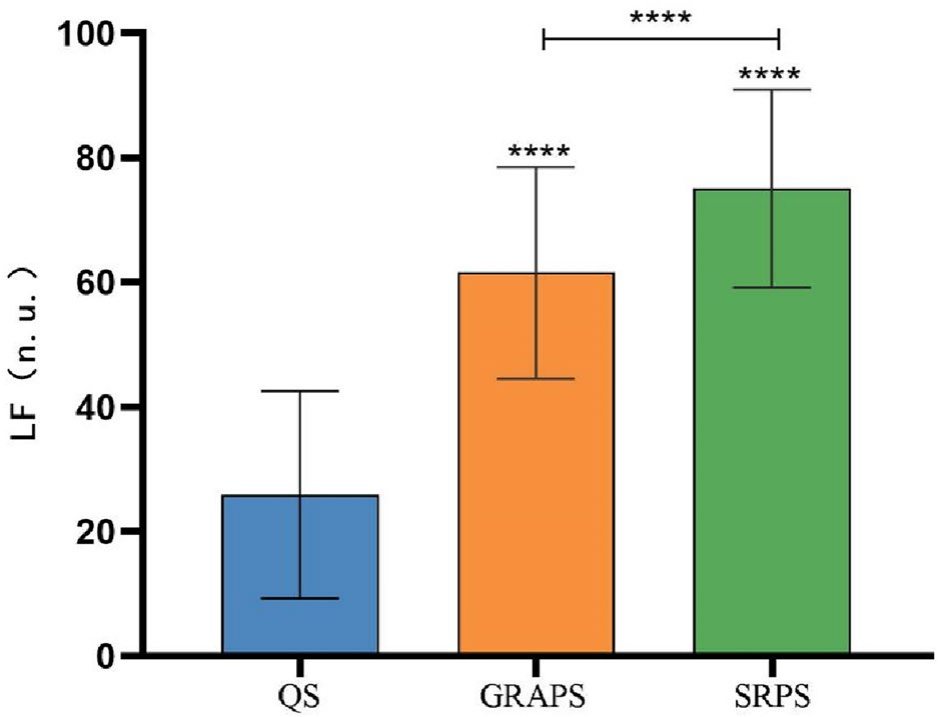
Schematic diagram of the LF (nu) comparison between the state of 24-form Tai Chi’s GRA protocol and SR protocol. Note: ****P < 0.05;the symbol "****" above each bar indicates a significant difference compared to the quiet state, *P* < 0.05;  the LF (nu) between the two groups is significantly different, *P* < 0.05.

As shown in Fig. 6, we conducted a repeated measures ANOVA to analyze the LF (nu) data across various states among Tai Chi practitioners. Mauchly's test of sphericity confirmed that the data met the Huynh–Feldt condition (*p* > 0.05), affirming the validity of the univariate ANOVA results. These results revealed a significant main effect over time (*P* < 0.01).

Significant disparities (*P* < 0.05) in LF (nu) were evident among different states, including the quiet state (25.92 ± 16.64 ms), the GRA protocol state (61.54 ± 16.94 ms), and the SR protocol state (75.03 ± 15.92 ms). To delve further into the distinctions between the GRA protocol state and the SR protocol state, we conducted a post hoc pairwise comparison. The findings indicated that LF (nu) in the GRA protocol state was significantly lower than that in the SR protocol state (*P* < 0.05).

In a separate analysis, a repeated measures ANOVA was performed on LF (nu) data from different states of Tai Chi practitioners. Mauchly's test of sphericity indicated a violation of the Huynh–Feldt condition (*P* < 0.05), necessitating the use of the Greenhouse-Gsiser estimate to correct for degrees of freedom. This correction revealed a significant main effect over time (*P* < 0.05).

**Figure 7:**
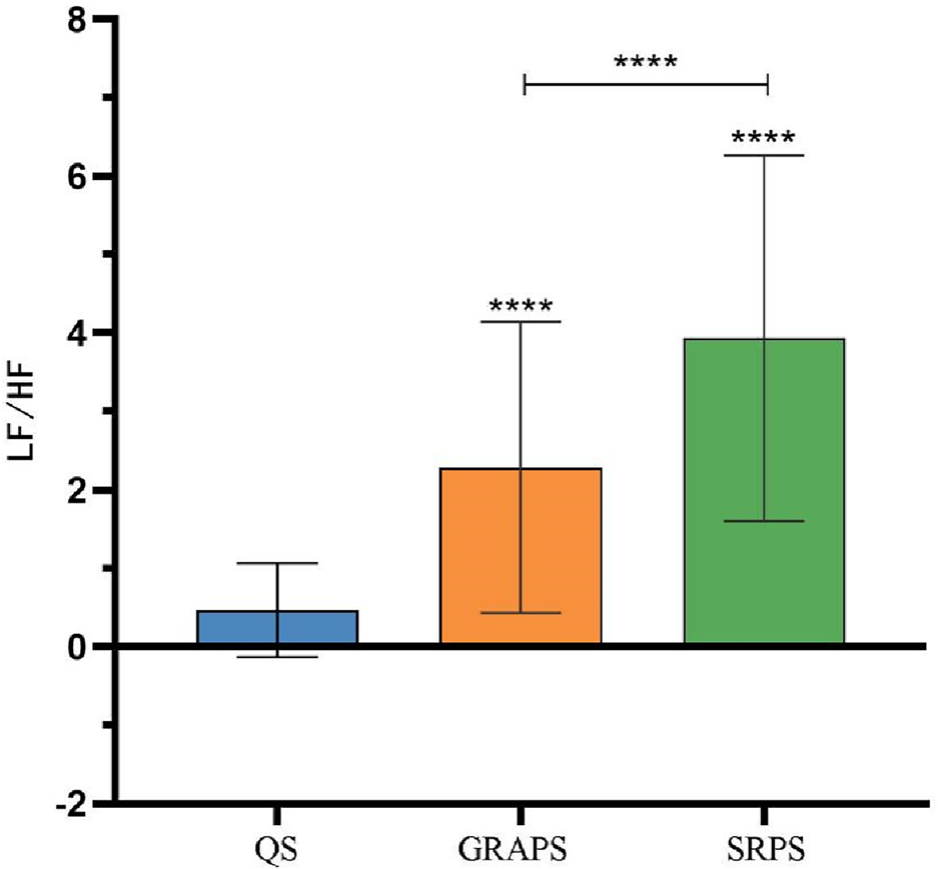
Schematic diagram of the LF/HF comparison between the state of 24-form Tai Chi’s GRA protocol and SR protocol. Note: ****P < 0.05;the symbol "****" above each bar indicates a significant difference compared to the quiet state, *P* < 0.05;  the LF/HF between the two groups is significantly different, *P* < 0.05.

As shown in Fig. 7**,** a repeated-measures ANOVA was employed to analyze the LF/HF data across various states among Tai Chi practitioners. Mauchly's test of sphericity indicated a violation of the Huynh–Feldt condition (*P* < 0.05), necessitating the utilization of the Greenhouse-Gsiser estimate to adjust for degrees of freedom. Following this correction, a significant main effect over time was observed (*P* < 0.05).

### The heart rate and heart rate variability data between each section of the GRA

**Figure 8:**
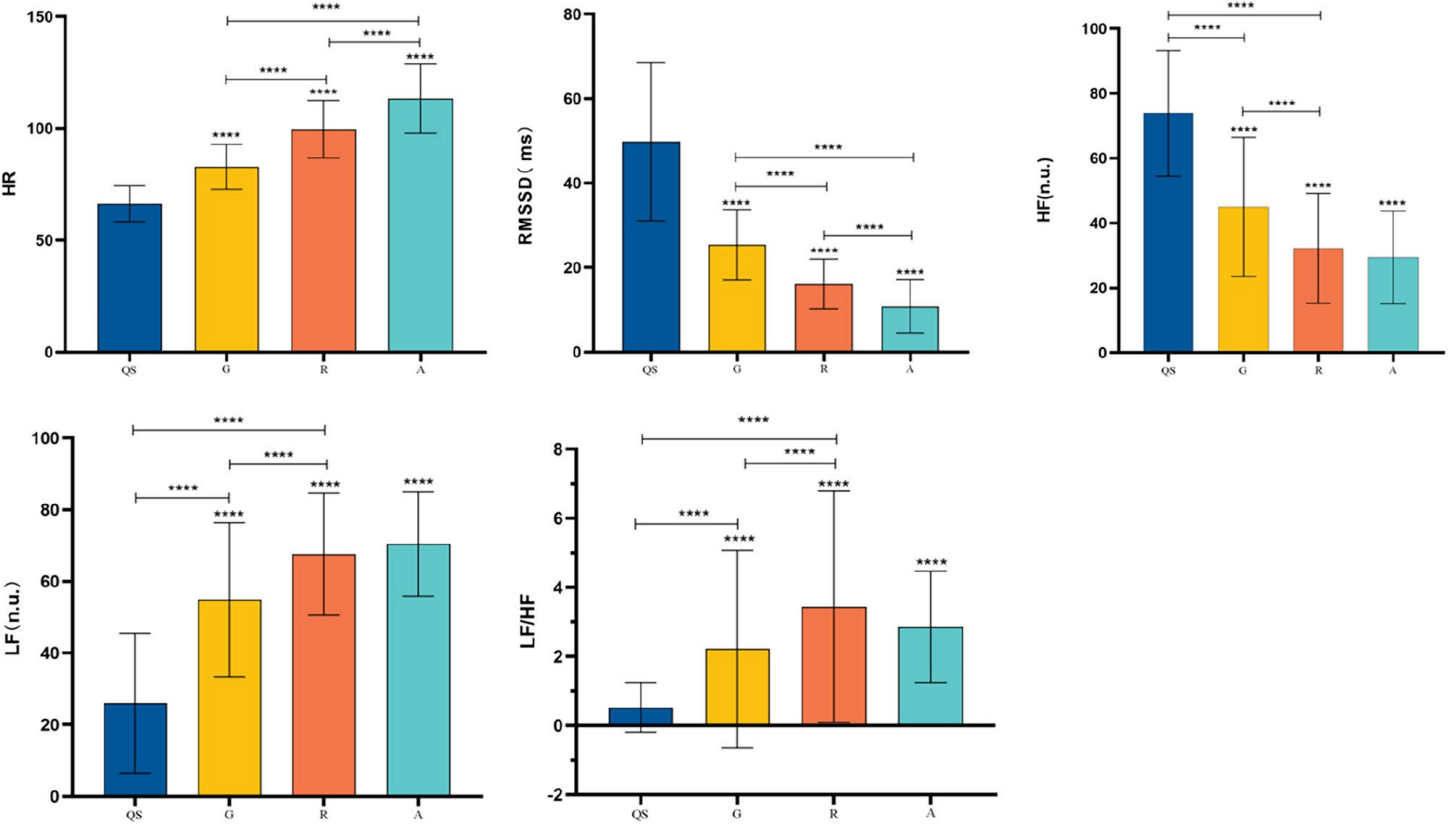
Schematic diagram of the Heart Rate and Heart Rate Variability comparison between each stage of the GRA protocol. Note: ****P < 0.05;the symbol "****" above each bar indicates a significant difference compared to the quiet state, *P* < 0.05;  the indicates between the two groups is significantly different, *P* < 0.05.

As shown in Fig. 8**,** a repeated-measures ANOVA was employed to analyze the data across 4 sections(Qiuent State, Gong Fa, Routine, and Application) among Tai Chi practitioners. Mauchly's test of sphericity indicated a violation of the Huynh–Feldt condition (*P* < 0.05), necessitating the utilization of the Greenhouse-Gsiser estimate to adjust for degrees of freedom. Following this correction, a significant main effect over time was observed (*P* < 0.05).

There were significant differences in heart rate and heart rate variability indexes, and the autonomic load showed a "stepped" upward trend in Tai Chi practitioners during the protocols of gong fa, routine, and self-defense application. Heart rate during self-defense application was the highest (113.4 ± 15.61 beats/min), significantly higher than that of the routine (99.69 ± 12.84 beats/min), gong fa (82.80 ± 10.60 beats/min), and quiet state (66.31 ± 8.01 beats/min). Time domain: self-defense application had the lowest RMSSD (10.78 ± 6.34 ms), significantly lower than routine (16.13 ± 5.93 ms), gong fa (25.38 ± 8.36 ms), and quiet state (49.85 ± 18.75 ms). Frequency domain: the self-defense application has the lowest HF (nu) (29.44 ± 14.31 ms) and the highest LF (nu) (70.42 ± 14.49 ms).

## Discussion

This study delves into the disparities in autonomic load between the GRA protocol and the SR protocol within the context of 24 form Tai Chi. The primary objective of this research is to furnish evidence pertaining to autonomic load, thereby informing the selection of practice methods for Tai Chi health promotion interventions. The study's findings indicate that the GRA protocol is associated with reduced autonomic stimulation compared to the SR protocol. Furthermore, the study suggests that the physiological stimulus stemming from heart rate elevation during Tai Chi practice exerts a more pronounced influence on autonomic activity when juxtaposed with psychological factors.

### Heart rate

Several studies have suggested that Tai Chi Quan falls within an intensity range of 52–63% of HR max, which aligns it with low-to-moderate-intensity aerobic exercises^30^. Notably, within the context of Tai Chi practice, heart rate levels are significantly lower during the GRA protocol compared to the SR protocol. Examining the movement content, the high-intensity exercises within the GRA protocol are primarily concentrated in the final 5 min, specifically within the "sparring" movements, imposing a substantial load during this phase. Conversely, the SR protocol imposes considerable demands on practitioners during leg control movements involving stirrups and independent downward momentum movements. Moreover, these intense movements need to be repeated thrice, rendering them dense and rigorous^31^. Continuous uninterrupted exercise is more cardiovascularly stimulating, more intense, and higher the heart rate^32^. In contrast, the GRA protocol incorporates intervals, including transitions from single to pair protocols and interruptions between movements in pair exercises. The SR protocol, on the other hand, entails a continuous practice of movements that are more strenuous than those in the GRA protocol. To sum up, both in terms of exercise content and duration, the SR protocol exhibits an overall higher exercise intensity compared to the GRA protocol.

### State of mind

In the context of the GRA protocol, Tai Chi practitioners exhibited significantly higher scores for agitation and activity while concurrently displaying significantly lower scores for calmness compared to the SR protocol. This difference can be attributed to the distinct nature of these practice protocols. Confrontational sports are more likely to be emotionally stimulating^33^. The GRA protocol involves elements of competitive and confrontational behavior, serving as a platform to hone the technical aspects of boxing movements. This confrontational component tends to stimulate heightened activity and excitement among practitioners. Mind–body exercises keep people in a quiet mood^26^. In contrast, the SR protocol entails continuous solo routines characterized by a focused mind and a relaxed physical and mental state, which naturally tilts the emotional disposition towards calmness. It's important to note that the confrontational content of the GRA protocol may be more emotionally stimulating and engaging when compared to the serene and tranquil nature of the SR protocol.

### Heart rate variability

The LF/HF ratio serves as an indicator of sympathetic modulation by reflecting the balance between sympathetic and parasympathetic nervous activities^34^. An increase in the LF/HF ratio signifies an imbalance favoring sympathetic nerve activity^35^. In this study, the results consistently indicated sympathetic dominance during exercise, with significantly higher sympathetic dominance observed in the SR protocol compared to the GRA protocol. It's worth noting that LF (nu) values, influenced by both sympathetic and parasympathetic nerves but more sensitive to sympathetic activity, were lower in the GRA protocol, signifying lower sympathetic activity in this protocol^36^. Conversely, the HF (nu) and RMSSD values in the GRA protocol were higher than those in the SR protocol, indicating enhanced parasympathetic nerve modulation in the former^37^.

Tai Chi practice influences autonomic activity through a combination of physiological and psychological factors^38^. Physiological load, such as cardiovascular stimulation, appears to exert the primary influence on the body's state, with psychological factors playing a secondary role^39^. Exercise intensity increasing, for instance, leads to an increased heart rate due to reduced parasympathetic activity and increased sympathetic activity^40^. In terms of heart rate, the SR protocol resulted in higher heart rates compared to the GRA protocol, with associated higher sympathetic activity and lower parasympathetic activity in the former. Mood elevation and psychological stress are typically associated with heightened sympathetic nervous activity, while relaxation correlates with greater parasympathetic nervous activity^41^. High heart rate variability is often linked to increased parasympathetic activity^42,43^.

Interestingly, despite psychological factors suggesting that the SR protocol should exhibit higher parasympathetic and lower sympathetic activity than the GRA protocol due to its higher calmness scores, the study's results showed the opposite protocol. This discrepancy suggests that physiological intensity may have a more significant impact on autonomic activity during Tai Chi exercises than psychological factors.

Cardiovascular stimuli and psychological influences impact autonomic nerves through distinct pathways^44^. In the case of exercise, stimulation of the heart activates physical and chemical receptors in the body's internal organs^40^. These receptors transmit nerve impulses to higher brain centers, leading to the activation of glutamatergic and gamma-aminobutyric nerves in specific regions like the nucleus solitarius, nucleus suspensus, ventral lateral nucleus of the medulla oblongata, and ventral lateral nucleus of the medulla caudalis^45^. This cascade ultimately results in an increase in norepinephrine secretion in the sympathetic ganglion, leading to heightened sympathetic activity and reduced parasympathetic activity^46^. Essentially, the physiological intensity stemming from cardiovascular stimuli affects autonomic nerves from the effector organs to the central nervous system and subsequently to the autonomic nerves^47^. On the other hand, certain studies suggest that intentional meditation activities, like those found in Tai Chi, stimulate specific brain areas such as the left anterior cingulate cortex, medial prefrontal cortex, and medial temporal lobe, among others, which are involved in regulating emotions^48,49^. This stimulation leads to changes in signal transduction substances, resulting in an increase in parasympathetic activity and a decrease in sympathetic activity^50^. This implies that the influence of psychological factors on autonomic activity follows a top-down pattern, originating from the central nervous system and reaching the autonomic nerves. However, during Tai Chi exercises, the calm emotional response induced by the SR protocol alone may not be sufficient to suppress sympathetic activity. Instead, the cardiovascular stimulation during sustained Tai Chi exercise appears to exert a more pronounced influence on autonomic nerves, resulting in significantly higher sympathetic activity compared to the GRA protocol.

### Different components of the GRA protocol

Study findings indicated a progressive increase in autonomic load during the GRA protocol. The self-defense application exhibited the highest sympathetic stimulation and the lowest in the gong fa. Firstly, the cumulative exercise load resulted in heightened excitatory sympathetic activity with prolonged exercise duration^40^. Secondly, in terms of content arrangement, self-defense application, with its confrontational practice content, induces greater sympathetic nerve activity and inhibits parasympathetic nerve activity. In gong fa practice, the content is designed to induce a state of tranquillity, facilitating physical and mental relaxation for the practitioner. Consequently, sympathetic activity is comparatively lower in gong fa practice when compared to routine and self-defense applications.

Additionally, it's crucial to note that the study participants primarily comprised college students, leading to the inclusion of falling exercises in the self-defense application part. The primary objective of incorporating falling exercises in the self-defense application is to know the participants' accuracy and proficiency in grasping the essence of Tai Chi movements. For frail elderly individuals, the self-defense application can be modified to focus on completing the action, inducing a shift in the opponent's center of gravity. Modifying the self-defense application allows for achieving a level of safety equivalent to the SR protocol. Initial attempts with this modification in the elderly population have been well-received. The application exercises provide stronger stimulation of proprioceptive and tactile senses in frail elderly individuals compared to the SR protocol. The GRA protocol holds potential for older individuals to attain enhanced health benefits, particularly in terms of fall prevention. Naturally, additional trials are required to substantiate this concept. In specific instances, the content of the self-defense application is adjusted to give the impression of losing center of gravity without actually causing a fall, especially in frail older adults and adults with mobility issues. Tai Chi beginners typically prioritize standing pole exercises (gong fa) initially, progressively allocating more time to routine practice, and eventually incorporating self-defense application. Adjusting the proportion of time dedicated to self-defense application exercises can be done both over the course of the training regimen and within a single session. This approach aids adults who are frail or have limited mobility in acquiring a deeper understanding of the form and essence of Tai Chi skills.

## Conclusion

The findings suggest that the intensity of cardiovascular stimulation during 24-form Tai Chi practice plays a more critical role in influencing autonomic activity compared to psychological factors. Specifically, heart rate levels are higher in the SR protocol than in the GRA protocol. This is accompanied by increased sympathetic activity in the SR protocol and decreased parasympathetic activity. The findings are limited to college students, and more research may be needed to provide support for frail older adults and adults with limited mobility issues. In light of these observations, it is advisable to make thoughtful choices when designing Tai Chi health promotion intervention programs. During the teaching phase, employing the GRA protocol can provide a low autonomic intensity for beginner practitioners to grasp the technical intricacies of Tai Chi. Combining the GRA protocol with the SR protocol allows Tai Chi practitioners to enhance their understanding of both the internal and external dimensions of Tai Chi skills. This approach acknowledges the varying impacts of different Tai Chi methods on autonomic activity and tailors the practice regimen accordingly. In addition, it is recommended that suggest more robust (experimental), larger trials to corroborate the findings.

## Data Availability

Data are available upon reasonable request from the corresponding author.
